# Arch-last technique for total arch replacement using two-pump system in Stanford type A aortic dissection

**DOI:** 10.1186/1749-8090-8-S1-P9

**Published:** 2013-09-11

**Authors:** C Park, C Choi, Y Jeon, J Lee, K Park

**Affiliations:** 1 Department of Thoracic and Cardiovascular Surgery, Gachon University, Gil Hospital, Incheon, Korea

## Background

Various cerebral protection methods are employed for aortic arch reconstruction. However, longer operation time and cerebral, cardiac and distal ischemic time impose a heavy burden on surgeons. This study demonstrates the clinical results and neurologic outcome with a modified cardiopulmonary bypass and antegrade cerebral perfusion in Stanford type A aortic dissection.

## Methods

Between June 2005 and December 2012, 22 patients (16 men and 7 women), aged 28 to 76 years(mean 56.7±13.8), underwent total aortic arch replacement using antegrade selective cerebral perfusion through median sternotomy. Present pathologies were Stanford type A dissection (acute state in 19, chronic state in 3 patients), previous Bentall's procedure in 3 and previous descending thoracic aortic repair in two patients. Operations were performed with antegrade cerebral perfusion through two options: with right axillary artery, left common carotid artery and left subclavian artery, or without the latter. After anastomoses of descending aorta and proximal ascending aorta operation were performed, the three neck vessels were each anastomosed during heart-beating with two pumps which were used for both cerebral and distal perfusion.

## Results

The mean duration of aortic clamping was 118±57 min. The mean duration of circulatory arrest of the lower body and cerebral perfusion was 54 ± 23 min. The postoperative average time of orientation recovery was 9.5 ± 7.5 hours. There were 2 patients of in-hospital mortality and 1 late death and no permanent neurologic deficit. Two patients underwent a staged reoperation for aneurysmal dilatation of residual descending aorta.

## Conclusion

The arch-last reconstruction with antegrade cerebral perfusion had good neurological results, provided shortened ischemic time of heart and distal lower body. This would be an alternative strategy for less experienced surgeon.

